# 

**Published:** 1816-09

**Authors:** 


### CATALOGUE OF MEDICAL BOOKS.

Mr. ACCUM has in the press, "A practical Essay on Chemical Re-agents or Tests, illustrated by a Series of Experiments." The work will comprehend a summary view of the general nature of chemical tests; the effects which are produced by the action of these bodies; the particular cases to which they may be applied, in the pursuits of chemical science; and the art of applying them successfully. A list of all those substances for which there exist any appropriate tests, will be added; and a portable chemical chest, containing all the chemical re-agents and apparatus necessary for performing the experiments described in the treatise, may also be had with the work, which will be published the 3d of this month.

Speedily will be published, Tables exhibiting the Division of the Body, Functions, and Organs; Distribution of Organs; Division and Bones of the Skeleton; Muscles of the Skeleton, of the Organs, Arteries, Veins, Absorbents, and Nerves. By T. J. Armiger, Member of the Royal College of Surgeons in London, Surgeon Extraordinary to H. R. Highness the Duke of Kent, and Surgeon to the Eastern Dispensary; Honorary Member of the Philosophical Society, and Member of the Medico-Chirurgical Society; late Demonstrator of Anatomy at the London Hospital.

Mr. HENRY ST. JOHN NEALE is preparing for the press, a new edition of Medical Essays and Practical Dissertations on the Nature, Causes, Symptoms, Treatment, and Cure, of the Tabes Dorsalis; with a variety of new Cases, and including Observations on Strictures in the Urethra.

Oracula Communications, addressed to Students of the Medical Profession; by ESCULAPIUS; is in the press.

---

AN Anterior and Posterior View of the Blood-Vessels, Nerves, and Muscles, of the Human Leg and Foot; coloured according to Nature.

Anecdotes, Medical, Chemical, and Chirurgical: collected, arranged, and transmuted, by an Adept. 2 vols. 12mo.

Elementary Principles of Physiology: translated from the French of Professor Majendie. Part I. 8vo.

A Narrative of a Journey to London, in 1814; or a Parallel of the English and French Surgery, preceded by some Observations on the London Hospitals. By P. J. Roux, M.D. 2d edition, translated from the French.

An Introduction to Comparative Anatomy and Physiology; being the two Introductory Lectures delivered at the Royal College of Surgeons, on the 21st and 25th of March, 1816, by William Lawrence, F.R.S. &c. 8vo.

Practical Observations on Typhus, and other Febrile Diseases. By John Armstrong, M.D. 8vo.

---

### *NOTICES TO CORRESPONDENTS.*

*We are much obliged to* J. M. A. *for reminding us of our promise, which shall be immediately attended to. In our own behalf, we are obliged to add, that, on inquiry, we found more mystery attached to the subject than we expected: however, we will make a point of gaining all the information we can, and of publishing all we learn.*

*We are favoured with Communications from* Dr. COLLINGWOOD, Dr. JOHN WALKER, Mr. SHATH, Mr. LITTLETON, *and* Mr. WALKER, *which, with several foreign Communications, shall appear in our next.*

*We have been honoured with several copies of new Publications, to which due respect shall be paid.*

